# Removal of most frequent microplastic types and sizes in secondary effluent using Al_2_(SO_4_)_3_: choosing variables by a fuzzy Delphi method

**DOI:** 10.1038/s41598-023-47803-4

**Published:** 2023-11-25

**Authors:** Nahid Azizi, Meghdad Pirsaheb, Nematollah Jaafarzadeh Haghighi, Ramin Nabizadeh Nodehi

**Affiliations:** 1 Department of Environmental Health Engineering, School of Public Health, Tehran University of Medical Sciences, Tehran, Iran; 2 Research Center for Environmental Determinants of Health (RCEDH), Health Institute, Kermanshah University of Medical Sciences, Kermanshah, Iran; 3 Department of Environmental Health Engineering, Faculty of Health, Kermanshah University of Medical Sciences, Kermanshah, Iran; 4 Department of Environmental Health Engineering, School of Public Health, Ahvaz Jundishapur University of Medical Sciences, Ahvaz, Iran

**Keywords:** Chemical engineering, Environmental chemistry

## Abstract

Microplastics (MPs) as an emerging pollutant can affect aquatic organisms through physical ingestion, chemical problems and possible creation of biological layers on their surfaces in the environment. One of the significant ways for MPs to enter the aquatic environment is through the effluent discharge of wastewater treatment plants (WWTPs). In this study, first, the concentration and characteristics of MPs in secondary wastewater effluent, and the influential variables related to the coagulation process, for MPs removal were identified using systematic reviews of previous studies. Then, the most proper MPs characterization and coagulation variables were chosen by experts’ opinions using a fuzzy Delphi method. Therefore, the experiment tested in conditions close to the full-scale wastewater treatments. Finally, in the laboratory removal of MPs by coagulation of polyamide (PA), polystyrene (PS), and polyethylene (PE), < 125 and 300–600 μm in size, was tested by a jar test applying Al_2_(SO_4_)_3_ in doses of 5 to 100 mg/L plus 15 mg/L polyacrylamide as a coagulant aid. Using R and Excel software, the results were analyzed statistically. It was concluded that the maximum and minimum removal efficiency was 74.7 and 1.39% for small PA and large PE, respectively. Smaller MPs were found to have higher removal efficiency. The MPs type PA achieved greater removal efficiency than PS, while PE had the least removal efficiency.

## Introduction

In recent years, technological advancements and the increase in prosperity have led to the increasing use of plastic materials. The countless properties of plastic, such as elasticity, cheapness, lightweight, and the convenience of using disposable plastic items led to the generation of 348 million tons of plastic waste, globally, in 2017 and will be increased four times by 2050^1–4^. The increase in these products' usage has subsequently led to a rise in the percentage of plastic materials in waste discharge and the production of microplastics (MPs) as an emerging pollutant. MPs are plastic materials smaller than 5 cm^5,6^ that exist in the soil, air, and water. Atmospheric MPs enter the soil through deposition and eventually fall into receiving waters through run-off. Another more significant way for MPs to enter the aquatic environment is through the effluent discharge of wastewater treatment plants (WWTPs)^7^. MPs can affect aquatic organisms in several ways. Among the problems of MPs is their physical ingestion, which creates false satiety and leads to starvation. The chemical problem is because of the absorption of dangerous chemicals, including additives in plastic, causing toxic leaks after entering the body of a living organism^5^. Another problem of MPs is the possible creation of biological layers on their surfaces in the environment, which become places for the accumulation of pathogenic microorganisms and can cause disease if they enter the body of a living organism^8,9^. Previous studies have investigated the profile of MPs in the different steps of conventional water and WWTPs^10^. At each step, the number of MPs decreases, but some of these particles have specific characteristics that were not significantly treated at different steps in conventional wastewater treatment plants^11^. Moreover, because of the high volume of wastewater effluent discharge in each treatment plant, there are numerous daily inputs of these substances to receiving water systems. Even though conventional WWTPs are an obstacle to the entry of MPs, they are still considered a point source for their entry into aquatic environments^12^.

Therefore, finding a suitable treatment for removing MPs from WWTPs seems necessary. In recent years, various methods have been suggested for removing these substances from aquatic environments, including membrane filters, absorption, ingestion by living organisms, biological decomposition, and coagulation^7,13,14^. Nonetheless, a simple and cost-effective process is necessary to be feasible for the full–scale removal of MPs. Among the various removal processes, the coagulation method, which has been used for particle removal in conventional treatment plants for many years, seems reasonable^5,15^. Shahi et al. investigated PE removal of 30–100 μm by AlCl_3_·6H_2_O and achieved 80% removal efficiency^16^. Lapointe et al. used both PE and PS 140 μm in size for their research and reported 82% and 84% removal efficiency, respectively^17^. Ma et al. chose a larger range of PE (less than 5 mm) and reported 61% removal efficiency^18^. Although these study results show that chemical coagulation can be a viable option for MPs removal^19^, the different types and sizes of MPs reported in WWTPs effluents have not been investigated. This process can be optimized as a proper tertiary full-scale treatment by choosing appropriate variables based on previous studies and experts’ opinions.

In this study, a systematic review of all articles published up to 2022 that targeted the profile of MPs in different steps of WWTPs was conducted, and the results were used to set the MPs concentration and characterization (types and sizes) to determine MPs removal efficiency by the coagulation process. In this novel approach, we tried to benefit from other studies and choose the best experimental set-up. Furthermore, to determine the proper variables of MPs removal by coagulation, articles that studied MPs removal by conventional coagulants in water and wastewater treatments were also systematically reviewed. Finally, based on the results of systematic studies and using environmental health experts' opinions with the fuzzy Delphi decision-making method, the variables for MPs removal by coagulation were selected, and experiments by jar test were conducted under laboratory conditions.

## Materials and methods

**Figure 1:**
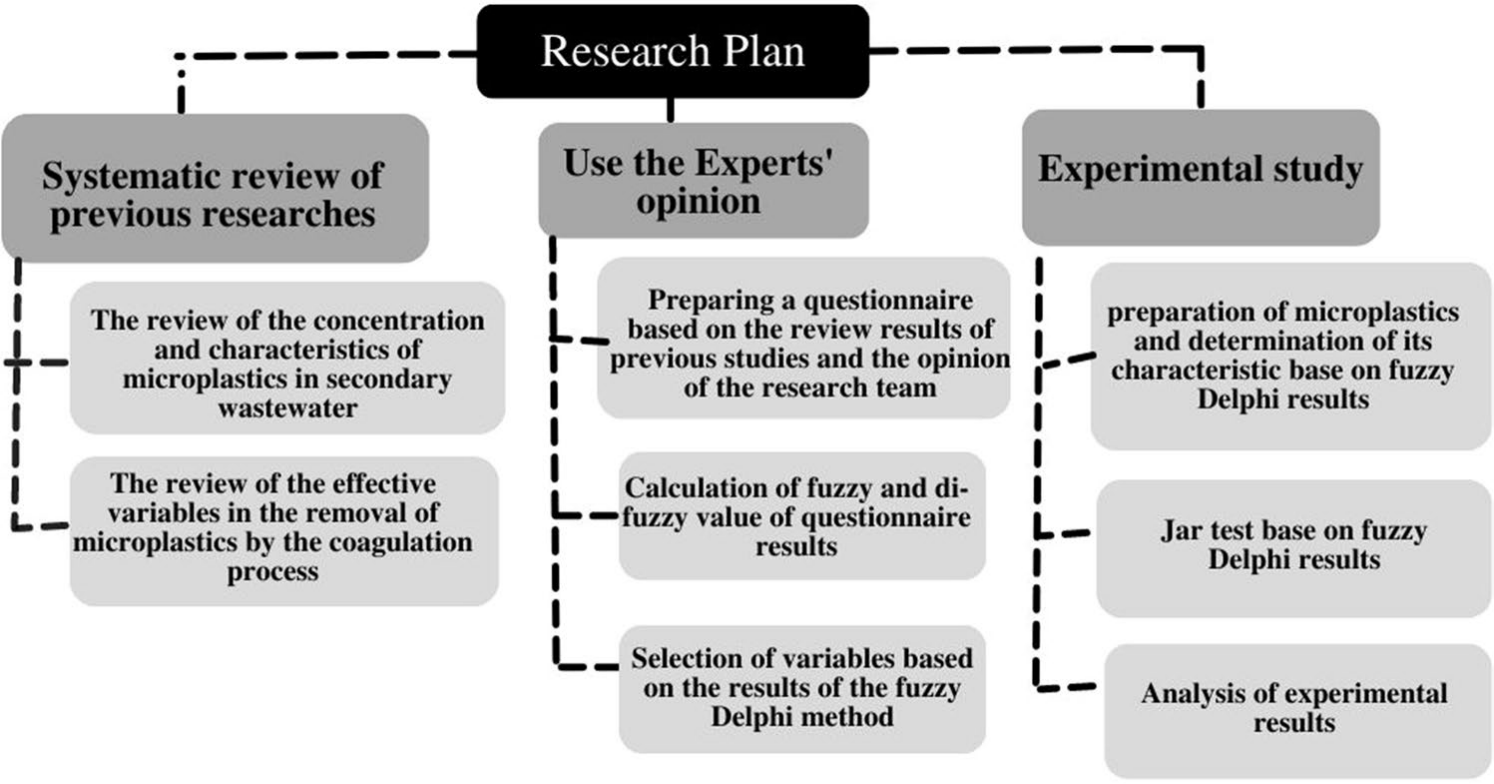
Organizational chart of the research plan.

The plan of this research to investigate MPs removal efficiency by the coagulation process is shown in Fig. 1. The first step was to perform a systematic review of previous research, including two systematic studies to determine the variables for MPs removal by coagulation. The first study, published in 2022 by Azizi et al.^11^, investigated the type and concentration of MPs in the secondary effluent of WWTPs. In the second study, previous articles were reviewed to determine the effective variables related to the coagulation process for MPs removal. In the next step, the experts' opinions were used to ultimately determine the variables based on the results of systematic studies through the fuzzy Delphi method. This step included questionnaire preparation and calculation of the fuzzy and di-fuzzy values to identify the proper variables. Finally, in an experimental study, the determined MPs were prepared and characterized; then, according to the variables obtained in fuzzy Delphi, MPs removal by coagulation was investigated through a jar test, and the results were analyzed. All the mentioned steps are detailed in the following sections. In addition, all methods were carried out in accordance with relevant guidelines and regulations, which were approved by the Tehran University of Medical Sciences licensing committee (Code of ethics: IR.TUMS.MEDICINE.REC.1399.1004).

### Systematic review results

A list of variables was created based on the review of previous articles, the results of WWTPs monitoring announced in^11^, and the results of articles related to the removal of MPs by coagulation (Table S2-1). Seven indices, comprising the location of the coagulation process in wastewater treatment; the concentration, type, and size of MPs; characteristics of the water matrix for conducting experiments; coagulant type; and use or non-use of enhanced coagulation were identified for designing the questionnaire.

### Use of experts’ opinions by fuzzy Delphi method

#### Identification and selection of expert panel members

First, experts were identified by the criteria-based method among the list of all scientific ranks of environmental health engineering in Iran, available in the scientific evaluation system of Iranian faculty members (https://isid.research.ac.ir/). Based on criteria such as having published or presented articles and teaching or work experience in the field of water and wastewater treatment, 35 experts were identified. A form contained the research topic, the purpose of the study, duration time, and approximate number of fuzzy Delphi courses, was designed and emailed to the 35 identified experts, and they were asked to express their willingness to participate in the panel of experts (participants)^20,21^.

#### Preparation of the initial questionnaire

The initial questionnaire contained 33 questions consisting of 23 Likert scale questions (strongly agree, agree, no opinion, disagree, strongly disagree), eight open questions, and two optional questions to cover all aspects of the identified variables (Table S1-3). Complementary comments were identified through open questions for use in the next courses. According to the analysis, only two courses were required. Calculations on the questionnaire, method of the validity and reliability check, agreement, and fuzzy value of each question are given in Supplementary File 1.

### Experimental study

All methods were carried out in accordance with relevant guidelines and regulations, all experimental protocols were approved by the Tehran University of Medical Sciences and informed consent was obtained from all subjects and/or their legal guardian(s).

#### Materials

Aluminum sulfate (Al_2_(SO_4_)_3_), polyacrylamide (PAM), sodium hydroxide (NaOH), and hydrochloric acid (HCl) were purchased from Merck Chemicals. 10-µm filter papers (F2040) were purchased from CHM. Hydrochloric acid was used to dissolve the coagulant attached to the MPs before filtration.

#### Preparation of MPs

Food-grade polystyrene cups were used to prepare polystyrene (PS) particles (density 1.05 g/cm^3^), and nylon cable was used for polyamide (PA) (density 1.13 g/cm^3^). A commercial blender (MODEL BB90E) was used to degrade the plastics to the desired size. In addition, polyethylene (PE) powder was purchased from a local plastic manufacturing plant. MPs particles were sieved with 30-, 50-, and 120-mesh (pore size 600, 300, and 125 μm) sieves and classified into two sizes: less than 125 μm (small), and 300 to 600 μm (large). The nature of the produced MPs was confirmed by Fourier Transform Infrared Spectroscopy (FTIR) (Fig. S2-1).

#### Jar test

To carry out the coagulation process, the Lovibond jar test machine, Germany (with six stirrers) was used. At the suggestion of the experts through Fuzzy Delphi, common coagulants were checked by pretest conducted in the laboratory, and (Al_2_(SO_4_)_3_) was confirmed for this study. Then, based on the chosen variables, the removal efficiency of MPs was evaluated.

#### MPs measurement

Many methods for detecting MPs in water have been investigated. However, no method has been found to be satisfactory because of the complexities of water quality^22^. The MPs used in this study floated on the water surface because their density was lower than that of water; therefore, the weight method was used to calculate the efficiency. Compared to current methods (microscopic, infrared methods, etc.), the weighing method is much more accurate^18,23^.

First, the MPs were placed in a Hotbox oven with a fan (GALLENKAMP, England) at 60 °C for 12 h to dehydrate. Then, dehydrated MPs were added to 500 mL of tap water in a 1-L beaker and weighed by a precision balance with a minimum weight range of 1 × 10^–5^ g (CP225D, SARTORIUS AG GÖTTINGEN, Germany) (W total). After settling for 30 min, the supernatant was carefully removed with a 50 ml syringe. In the next step, the MPs in the supernatant were immersed in 0.1 mol/L hydrochloride for 1 h to remove the coagulants and then passed through the filter using a vacuum pump. Finally, the MPs on the surface of the filter were carefully separated and washed inside a plate to be dried again in the drying air oven at 60 °C for 12 h. After cooling down to room temperature, they were weighed (W remained). Therefore, the percentage of removal efficiency can be expressed (by applying the coefficient of the sample volume) as follows:

$${\text{MP}}\;{\text{removal}}\, = \,\left[ {\left( {{\text{W}}_{{{\text{total}}}} {-}{\text{W}}_{{{\text{remained}}}} } \right)/{\text{W}}_{{{\text{total}}}} } \right]\, \times \,{1}00.$$

All experiments were repeated three times.

#### Quality assurance and quality control (QA/QC)

Before any test was performed, all necessary equipment was thoroughly euthanized (99%). A cotton coat and latex gloves were used to prevent contamination, and all tools used were made of glass. To detect particles in the air and/or general errors, a blank sample was always used along with other samples^23,24^.

#### Experiment design

To investigate the effect of the coagulation process based on the variables determined in fuzzy Delphi, a laboratory investigation of different Al_2_(SO_4_)_3_ concentrations (5, 10, 20, 30, 40, 50, 60, 70, 80, 90, and 100 mg/L) was conducted to remove three types of MPs PE, PS, and PA in small and large sizes (< 125 μm, and 300 to 600 μm, respectively). Sixty-six runs along with six blanks were performed and are shown in Table S2-2. Each run was repeated 3 times.

## Results and discussion

### Experts’ opinion in fuzzy Delphi

From the 35 experts sent questionnaires, 22 of them expressed a desire and willingness to participate in the research; their characteristics are presented in Table S2-3. The results and analyses related to the fuzzy Delphi method are given in supplementary file 1, and Table S2-4 shows the final Fuzzy and De-Fuzzy values related to each question.

### Choosing the variables of the process

As mentioned in a previous section, the systematic review revealed that the significant variables for the coagulation removal of MPs included MPs concentration and characteristics, pH, type and dose of both coagulants, and coagulant aid, time, and speed of mixing^25^.

#### MPs concentration and characterization

According to the experts’ opinion based on the results of the Delphi method, three MP types, namely PE, PS, and PA in the range of < 600 μm size in the secondary effluent concentration reported for WWTPs, were chosen for examination in this research, because these three MP types in the mentioned size range cannot be significantly removed in the conventional WWTPs steps. In addition, the small size of the MPs results in low accuracy when the coagulation removal process is performed at low concentrations. Therefore, the maximum concentration related to the secondary effluent (7,863 items per liter) reported in previous articles was used^11^.

#### pH, coagulant, and coagulant aid

Regarding pH and the type and concentration of the coagulant aid, 86% and 56% of the reviewed articles achieved optimal removal at pH 7 and using anionic polymer (15 mg/L), respectively^25^; thus, these amounts were used in the current research without the need of expert opinion. The experts recommended, however, that all coagulants used in previous studies be checked using the jar test and the most proper coagulant type chosen based on the results^25^. Based on the pretest results, Al_2_(SO_4_)_3_ was selected, and the coagulant dose was considered an independent variable in the laboratory phase^25^.

#### Settling and mixing time and speed

Previous studies have stated that 100 rpm is a very high speed which may prevent the growth of flocs or break flocs that have formed^19^.The present study investigated different speeds in the laboratory, and as the MPs floated on the surface of the water at lower speeds, the coagulant did not collide with the MPs. As a result, based on previous studies and the laboratory investigation, speeds of 300 and 100 rpm for 1 and 14 min were selected for slow and fast mixing, respectively. Most articles (79%) reported settling time as 30 min under optimal conditions, and this time was also used in this research.

#### Water solution

Regarding the selected matrix, Ma et al. found that the amount of turbidity and humic acid had a negligible effect on the removal of MPs^5^. Therefore, based on laboratory investigations, tap water was selected in this research to add to MPs in specific amounts. Each sample used 0.5 L of tap water, which gave the best mixing state in a beaker.

### Experimental* study*

#### Determination of MPs concentration

**Figure 2:**
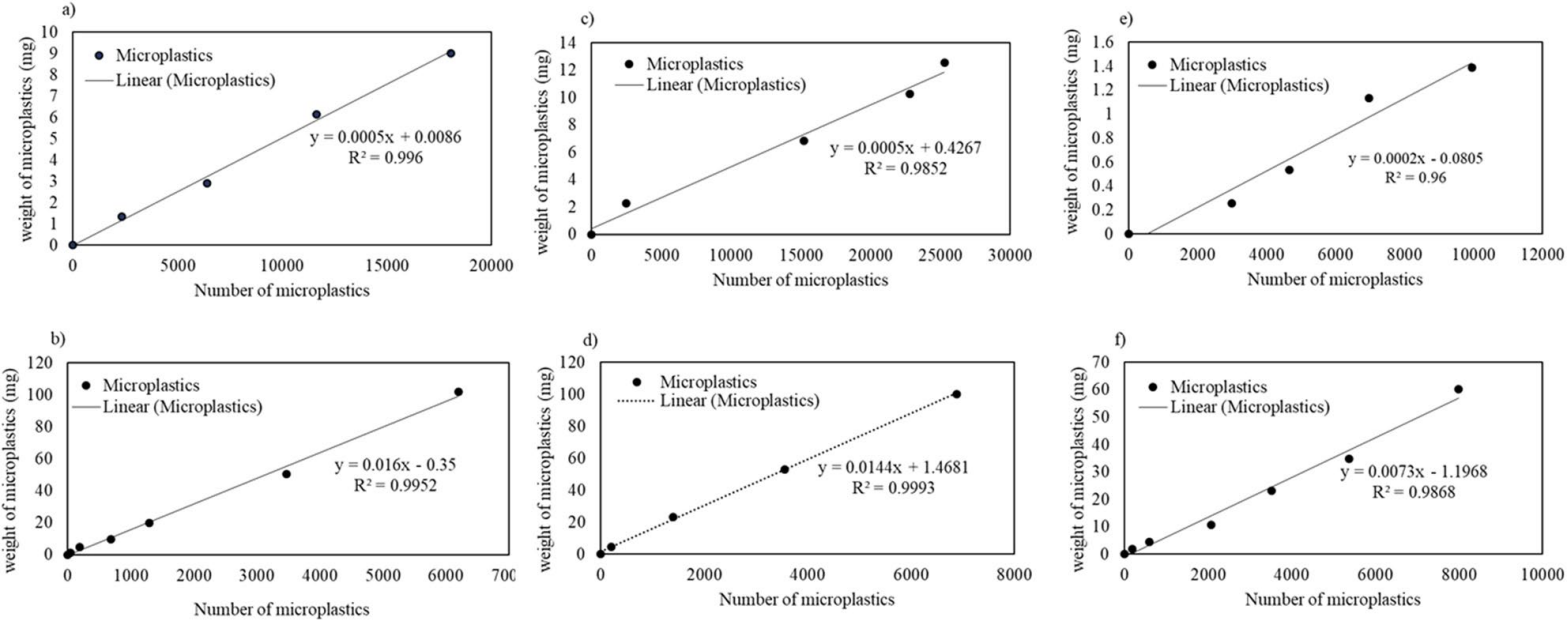
(**a**) PE < 125 µm (3.94 mg), (**b**) PE 300–600 µm (125.45 mg), (**c**) PS < 125 µm (4.35 mg), (**d**) PS 300–600 µm (114.69 mg), (**e**) PA < 125 µm (1.49 mg), (**f**) PA 300–600 µm (56.20 mg).

Because the articles reported MPs concentration as numbers per liter, and because counting each MP for each experiment is very time-consuming and reduces the accuracy of the measurement, the weight of the MPs was converted into a number by counting specific weight of MPs (both sizes of each type) using a Leica MS5 stereo microscope. Each weight was counted three times to estimate the counting error^26^. A model was designed for each MP (Fig. 2), and the related data of the models is given in supplementary file 3. The weight values obtained for each case (in terms of number per liter) are listed in the caption of Fig. 2. Conclusively, the initial concentrations of PE < 125, PE: 300–600, PS < 125, PS: 300–600, and PA < 125, PA: 300–600 were calculated as 3.94, 125.45, 4.35, 114.69, 1.49, and 56.2 mg, respectively.

#### Effects of coagulant dose on MPs removal efficiency

According to the linear regression analysis (Table 1), the size and type of MPs and coagulant doses have significant effects on removal efficiency (*p*-value < 0.05).

**Table 1:** Linear regression analysis results.

	Estimate	Std. error	t value	Pr( >|t|)
(Intercept)	35.95	5.02	7.15	1.25 × 10^–9^
Small size	43.89	3.89	11.27	< 2 × 10^–16^***
PE	− 39.61	4.76	− 8.30	1.31 × 10^–11^***
PS	− 24.47	4.76	− 5.13	3.15 × 10^–6^***
Coagulant dose	0.15	0.06	2.42	0.018*

Significant codes: 0 ‘***’ 0.01 ‘*’ 0.1 ‘’, Residual standard error: 15.82 on 61 degrees of freedom.

Multiple R-squared: 0.76, Adjusted R-squared: 0.75, F-statistic: 50.81 on 4 and 61 DF, p-value: < 2.2e−16.

**Figure 3:**
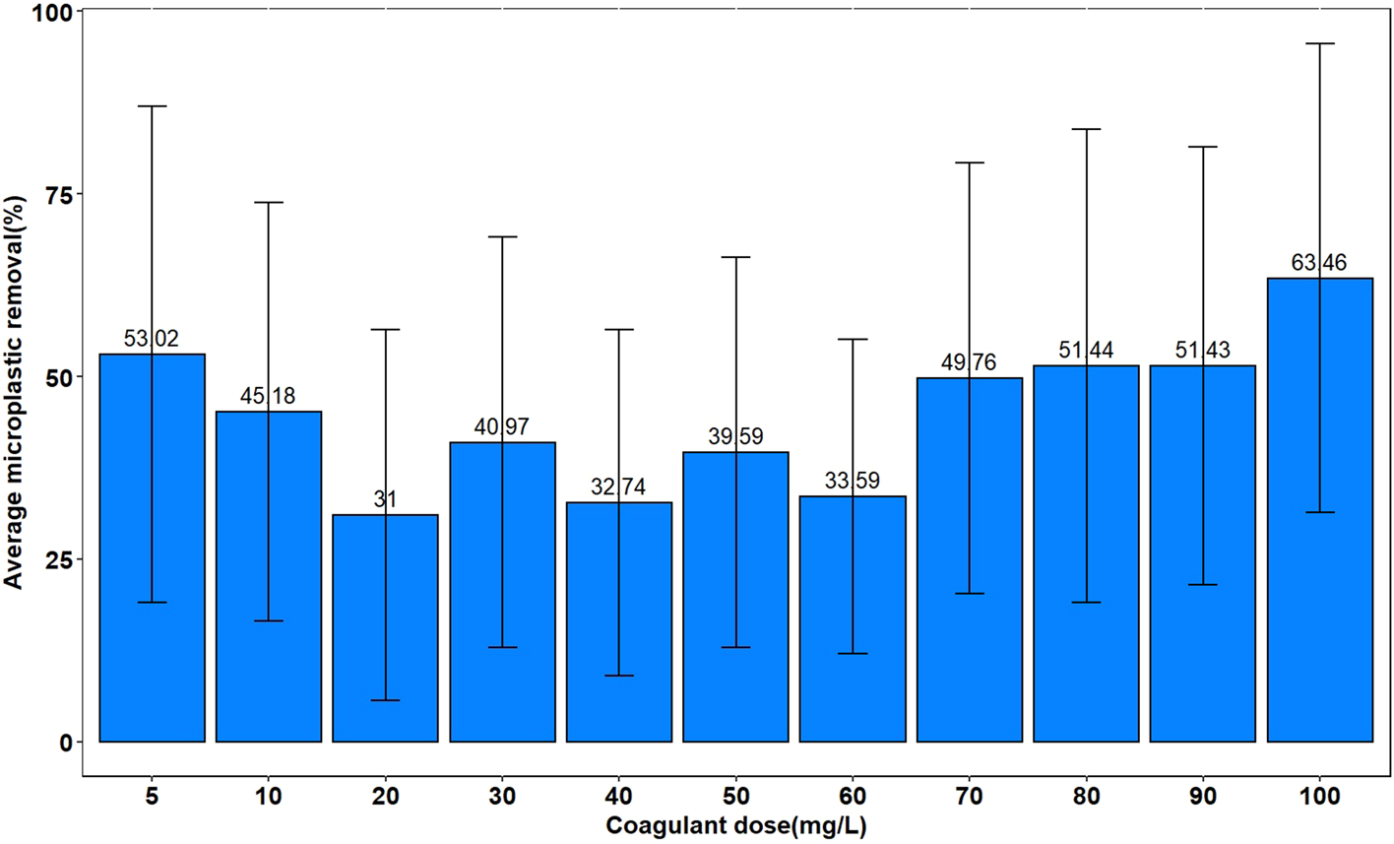
Removal efficiency in different coagulant doses.

To optimize the coagulant dose for each MP type, pH and PAM had fixed values of 7 and 15 mg/L, respectively, based on previous studies. The average removal efficiency of 3 types of MPs under different doses of Al_2_(SO_4_)_3_ is shown in Fig. S2-2. The results showed that the average removal efficiency ranged from 31 to 63% for 40 and 100 mg/L, respectively (Fig. 3).

As is clear from Fig. S2-2, the average removal efficiency in the two lowest coagulant doses of 5 and 10 mg for PA, PS, and PE was 74%, 51%, and 24%, respectively, which subsequently decreased with a further increase in dosage. This condition was because of the fact that larger MPs were partially settled without coagulant because of their higher weight, according to the results of the blank samples (Fig. S2-3). Therefore, even with the lowest floc formation, high efficiency was achieved. This phenomenon is less seen in the case of large PE, because PE particles are suspended in water, and the probability that they will settle naturally is very low. Thus, for these particles to settle requires floc formation, and the amount of PE removed by coagulation depends on the number of particles that are absorbed into the flocs^24^. On the other hand, increases in the concentration of the aluminum coagulant corresponded with increased aluminum hydroxide formation. As a result, fewer particles were captured in the clusters. Most of them are dispersed in water, which will lead to a decrease in efficiency^26^. With the dose of 60 mg/L, the removal efficiency of PA, PS, and PE decreased to 45, 40, and 21, respectively (Fig. S2-3). This may be explained by the increase in the surface-to-volume ratio of MPs by flocs, which causes them to float and reduces the amount of sedimentation, which subsequently reduces removal efficiency. By increasing the coagulant dose to 70, 80, and 90 mg/L, the rapid formation of flocs with greater cohesion and higher weight increased the sedimentation rate and removal efficiency^23^. However, the effect of coagulant dose on MP removal in the range of 70 to 90 mg/L was relatively smooth, and the increase in coagulant concentration had little impact on increasing efficiency; so for PA, PS, and PE, the average efficiency (± standard deviation) was 63 ± 3, 55 ± 0.5 and 34 ± 0.5%, respectively, and the enhancement of removal by increasing coagulant dose is not very clear. Finally, by further increasing the coagulant dose to 100 mg/L, the efficiency rates for PA, PS, and PE increased to 82%, 61%, and 46%, respectively which it was the optimal dose for Al_2_(SO_4_)_3_ (with average of 63%).

The use of the dissolved air flotation (DAF) method to remove PE particles was done without coagulation, and low efficiency (25% to 30%) was obtained. As a result, it can be said that coagulation and flocculation to remove MPs with high efficiency are necessary steps^24^. It can be said that because MPs float in water due to their low density, sufficient efficiency can be reached at a lower coagulant dose if the DAF method is used instead of sedimentation in the final stage of the coagulation process.

The coagulant dose in conventional water treatment is always less than 20 mg/L^24^. According to Table 2, the average removal efficiency for all MPs at this coagulant dosage was lower in the current study than in previous laboratory studies, while the removal efficiency rates of small-sized PA and PS separately at this dose were 79% and 68%, respectively, which is similar to other studies. Lapointe et al. and Shahi et al. used 4000 mg/L silica sand and 500 mg/L polyamine-coated (PC) sand, respectively, to improve removal efficiency. The higher efficiency achieved by Ma et al. can be attributed to the high concentration of MPs in lower sizes (200 mg/L). Moreover, the mentioned studies examined a single MP, but in WWTPs, much lower MP concentrations and a combination of MPs are found. It can be concluded that coagulation alone is not enough for the efficient removal of coagulants; it is better to use enhanced coagulation.

**Table 2:** Comparison of the current results and those of other coagulation studies.

Article	Microplastic	Size	Coagulant	Dose (mmol)	pH	Polymer	Polymer dose (mg/l)	Efficiency(%)
^17^	PE	100–500	Alum(Al(OH)_3_)	0.10	7	Anionic polymer	0.3	82
^17^	PS	100–500	Alum(Al(OH)_3_)	0.10	7	Anionic polymer	0.3	84
^5^	PE	100–500	AlCl_3_·6H_2_O	0.05	7	Anionic polymer	15	61.19
^16^	PE	5–100	AlCl_3_·6H_2_O	0.12	7	Polyamine	1	80.3
^16^	PE	5–100	AlCl_3_·6H_2_O	0.12	7	Polyamine	1	100
This study 1	PE,PS,PA	< 600	Al_2_(SO_4_)_3_	0.12	7	PAM	15	45.18
This study 2	PE,PS,PA	< 600	Al_2_(SO_4_)_3_	0.05	7	PAM	15	33.63
This study 3	PA	< 125	Al_2_(SO_4_)_3_	0.05	7	PAM	15	79
This study 4	PS	< 125	Al_2_(SO_4_)_3_	0.05	7	PAM	15	68

**Figure 4:**
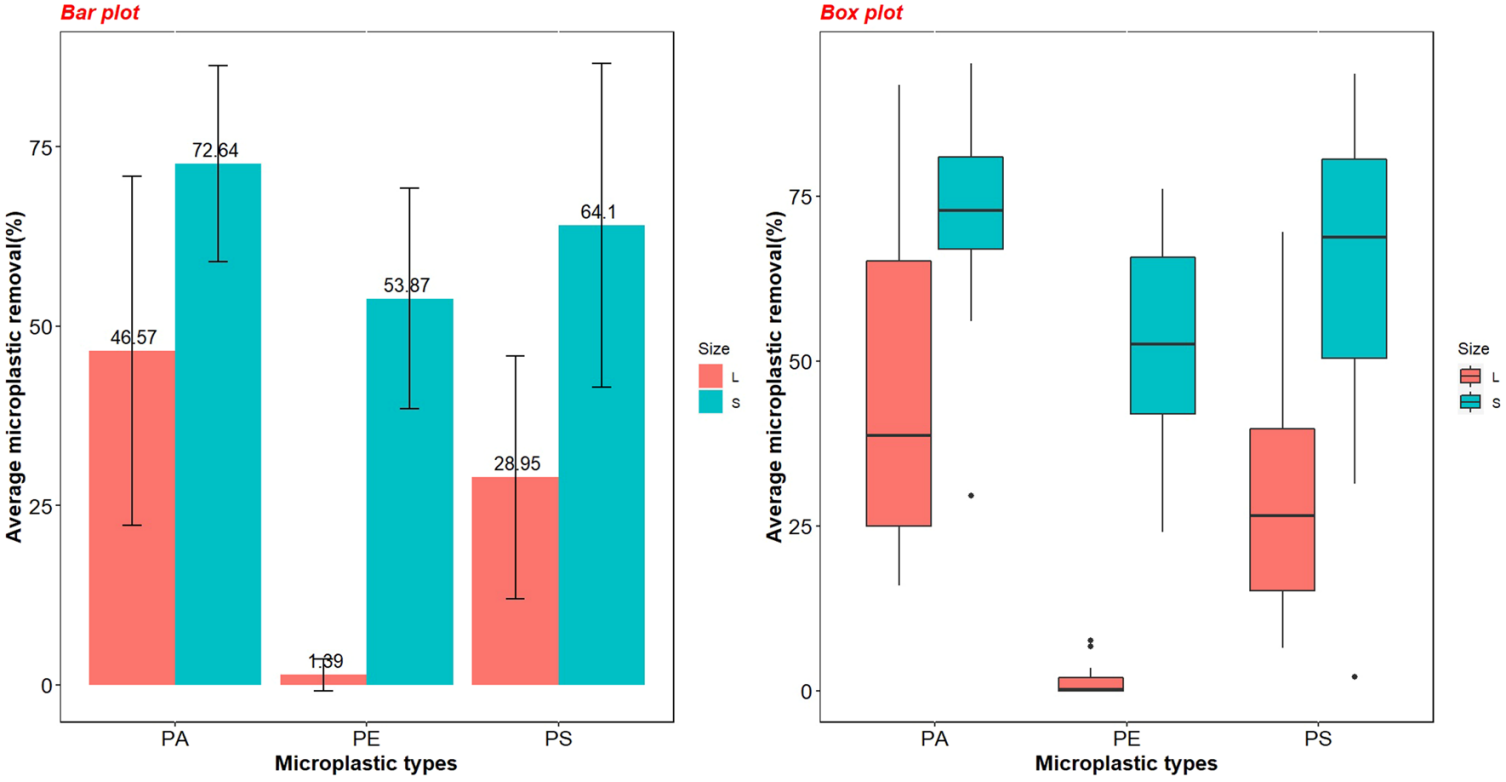
Average microplastic removal rates in different microplastic types and sizes.

**Figure 5:**
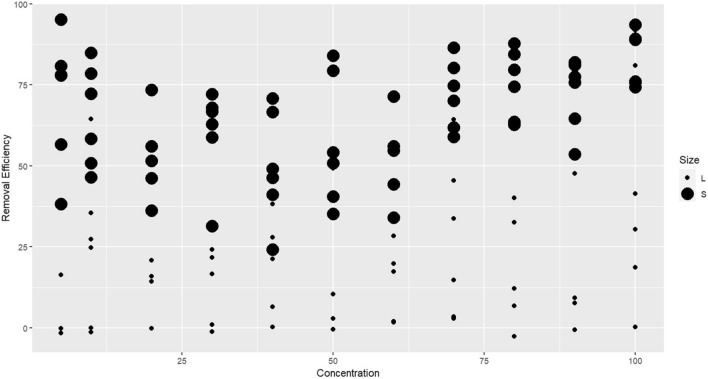
Removal efficiency in different coagulant dosages in case of microplasic size (S: MPs < 125, L: MPs = 300–600).

As can be seen in Table S2–5, in addition to coagulant dose, the size and type of MPs are effective variables in MPs removal. The power of ANOVA calculated for MPs type and size were 83.4% and 80.7%, respectively (Table S2–6). Based on the results of ANOVA (Table S2-5), the average removal efficiency of MPs < 125 μm was significantly higher (*p*-value < 0.05) than 600–300 μm MPs (averages of 65% and 22.5%, respectively). As shown in Figs. 4, 5 the higher efficiencies all belong to small MPs (< 125 μm) (showed by large black spheres) and are 53.8%, 64.1%, and 72.6% in average for PE, PS, and PA, respectively. In some cases, an efficiency greater than 90% was attained, while the majority of removal efficiency rates for larger MPs (300–600 μm) were below 25%, at 1.39%, 28.9%, and 46.5% in average for PE, PS, and PA, respectively. In previous studies, a higher dose used to remove larger MPs than smaller MPs, which is consistent with the current study^19^.

**Figure 6:**
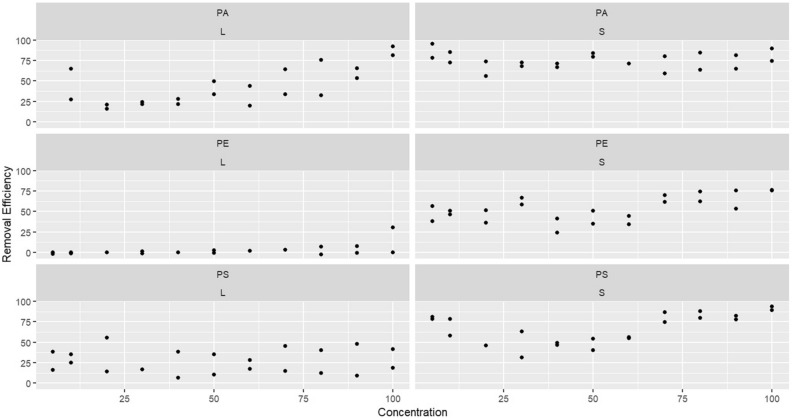
Microplastic removal by coagulant dose and microplastic type and size.

According to the ANOVA analysis performed for MPs type (Table S2-5), the removal efficiency was significantly higher for PA (the average of 59%) than PS and PE. Although the average removal of PS was higher than that of PE (averages were 47% and 28%, respectively); the difference, however, was not statistically significant (Table S2-7). In general, removal efficiency rates were 74.7%, 67%, 53.9%, 40.9%, 27%, and 1.39% for small PA, small PS, small PE, large PA, large PS, and large PE, respectively (Fig. 4). A relatively increasing trend for removal efficiency in large PA, PS, and small PE with increasing coagulant doses can be seen in Fig. 6, but the other cases showed a stable and smooth trend in all coagulant dosages. Large PE and PS in all coagulant dosages attained less than 25% and 50% removal efficiency, respectively, while large polyamide with the addition of 60 mg/L coagulant dose, reached a high efficiency of 60%. Nonetheless, the smaller MPs in the same dose reached a high efficiency of 75%.

Most studies that have investigated the mechanism of MPs removal by coagulation have concluded that the main mechanism is charge neutralization and sweeping^19,24,26^. According to laboratory observations, larger polyethylene MPs form clusters and float on water instead of settling because of their lower density^19^; as a result, their removal through sedimentation is less than the other two types; even zero efficiencies have been obtained in this category. Another factor of the low removal efficiency in PE can be stability because of the electrical transfer of negative colloids in water and excessive absorption of negative ions^23^. Small polyethylene particles form some flocs and temporarily disperse in water; then, a percentage of them settles over time, which leads to higher efficiency compared to larger particles with same type^26^.

## Conclusion 

In this study, the results of previous studies and experts' opinions were used in examining the variables related to removing MPs by the coagulation process. This process is a low-cost solution to improve the efficiency of tertiary systems and minimize the discharge of MPs through the effluent of WWTPs. The results show that the appropriate adaptation of conventional technologies like coagulation can sufficiently promote the removal of MPs from wastewater.

Based on the results of the experiment, on average, the removal efficiency was 74.7%, 67%, 53.9%, 40.9%, 27%, and 1.39% for small PA, small PS, small PE, large PA, large PS, and large PE, respectively. Because of the low-density nature of MPs, however, it can be suggested to use methods such as DAF that bring the flocs to the surface instead of the sedimentation method that has been used in previous researches as well as the current study.

Because in full-scale conditions, the number of particles in the effluent of the secondary treatment is much lower than the concentrations that have been used in laboratory cases, it seems that upgrading the process by adding particles such as sand can be a proper suggestion for future studies. Furthermore, additional methods such as filtration as a completion step can be useful for the complete removal of MPs. Plus, the consideration of the long-term effectiveness and potential environmental impacts of coagulation for full-scale experiments can be suggested.

### Supplementary Information

Supplementary Information 1.

Supplementary Information 2.

Supplementary Information 3.

## Data Availability

All data generated or analysed during this study are included in this published article [and its supplementary information files].

## References

[1] Herbort AF, Sturm MT, Fiedler S, Abkai G, Schuhen K (2018). Alkoxy-silyl induced agglomeration: A new approach for the sustainable removal of microplastic from aquatic systems. J. Polym. Environ..

[2] Ghayebzadeh M, Taghipour H, Aslani H (2020). Estimation of plastic waste inputs from land into the Persian Gulf and the Gulf of Oman: An environmental disaster, scientific and social concerns. Sci. Total Environ..

[3] OECD (2022). Global Plastics Outlook: Economic Drivers, Environmental Impacts and Policy Options.

[4] Tiwari R, Azad N, Dutta D, Yadav BR, Kumar S (2023). A critical review and future perspective of plastic waste recycling. Sci. Total Environ..

[5] Ma B, Xue W, Ding Y, Hu C, Liu H, Qu J (2019). Removal characteristics of microplastics by Fe-based coagulants during drinking water treatment. J. Environ. Sci..

[6] Ghayebzadeh M, Aslani H, Taghipour H, Mousavi S (2020). Estimation of plastic waste inputs from land into the Caspian Sea: A significant unseen marine pollution. Mar. Pollut. Bull..

[7] Nasseri S, Azizi N (2022). Occurrence and Fate of Microplastics in Freshwater Resources.

[8] Sun J, Dai X, Wang Q, van Loosdrecht MC, Ni B-J (2019). Microplastics in wastewater treatment plants: Detection, occurrence and removal. Water Res..

[9] Azizi N, Khoshnamvand N, Nasseri S (2021). The quantity and quality assessment of microplastics in the freshwater fishes: A systematic review and meta-analysis. Reg. Stud. Mar. Sci..

[10] Taghipour H, Ghayebzadeh M, Ganji F, Mousavi S, Azizi N (2023). Tracking microplastics contamination in drinking water in Zahedan, Iran: From source to consumption taps. Sci. Total Environ..

[11] Azizi N, Nasseri S, Nodehi RN, Jaafarzadeh N, Pirsaheb M (2022). Evaluation of conventional wastewater treatment plants efficiency to remove microplastics in terms of abundance, size, shape, and type: A systematic review and meta-analysis. Mar. Pollut. Bull..

[12] Talvitie J, Mikola A, Koistinen A, Setälä O (2017). Solutions to microplastic pollution—Removal of microplastics from wastewater effluent with advanced wastewater treatment technologies. Water Res..

[13] Baaloudj O, Nasrallah N, Kenfoud H, Bourkeb KW, Badawi AK (2023). Polyaniline/Bi_12_TiO_20_ hybrid system for cefixime removal by combining adsorption and photocatalytic degradation. ChemEngineering..

[14] Badawi AK, Salama RS, Mostafa MMM (2023). Natural-based coagulants/flocculants as sustainable market-valued products for industrial wastewater treatment: A review of recent developments. RSC Adv..

[15] Ramirez L, Gentile SR, Zimmermann S, Stoll S (2016). Comparative study of the effect of aluminum chloride, sodium alginate and chitosan on the coagulation of polystyrene micro-plastic particles. J. Colloid Sci. Biotechnol..

[16] Shahi NK, Maeng M, Kim D, Dockko S (2020). Removal behavior of microplastics using alum coagulant and its enhancement using polyamine-coated sand. Process Saf. Environ. Prot..

[17] Lapointe M, Farner JM, Hernandez LM, Tufenkji N (2020). Understanding and improving microplastic removal during water treatment: Impact of coagulation and flocculation. Environ. Sci. Technol..

[18] Ma B, Xue W, Hu C, Liu H, Qu J, Li L (2019). Characteristics of microplastic removal via coagulation and ultrafiltration during drinking water treatment. Chem. Eng. J..

[19] Rajala K, Grönfors O, Hesampour M, Mikola A (2020). Removal of microplastics from secondary wastewater treatment plant effluent by coagulation/flocculation with iron, aluminum and polyamine-based chemicals. Water Res..

[20] Ahmadi M, Zakerian SA, Salmanzadeh H (2017). Prioritizing the ILO/IEA Ergonomic Checkpoints' measures; a study in an assembly and packaging industry. Int. J. Ind. Ergon..

[21] Ahmadi M, Zakerian SA, Salmanzadeh H, Mortezapour A (2016). Identification of the ergonomic interventions goals from the viewpoint of ergonomics experts of Iran using Fuzzy Delphi Method. Int. J. Occup. Hyg..

[22] Li J, Dagnew M, Ray MB (2022). Effect of coagulation on microfibers in laundry wastewater. Environ. Res..

[23] Zhang Y, Zhao J, Liu Z, Tian S, Lu J, Mu R (2021). Coagulation removal of microplastics from wastewater by magnetic magnesium hydroxide and PAM. J. Water Process Eng..

[24] Esfandiari A, Mowla D (2021). Investigation of microplastic removal from greywater by coagulation and dissolved air flotation. Process Saf. Environ. Prot..

[25] Azizi N, Pirsaheb M, Jaafarzadeh N, Nodehi RN (2023). Microplastics removal from aquatic environment by coagulation: Selecting the best coagulant based on variables determined from a systematic review. Heliyon..

[26] Skaf DW, Punzi VL, Rolle JT, Kleinberg KA (2020). Removal of micron-sized microplastic particles from simulated drinking water via alum coagulation. Chem. Eng. J..

