# Supplementary material for: Removal of most frequent microplastic types and sizes in secondary effluent using Al2(SO4)3: choosing variables by a fuzzy Delphi method
Source: Sci Rep. 2023 Nov 25;13:20718. doi: 10.1038/s41598-023-47803-4 (PMC10676421; doi:10.1038/s41598-023-47803-4)
Supplement: Supplementary file 1 — Supplementary Information 1. [file 41598_2023_47803_MOESM1_ESM.docx]

# Supplementary file 1

**Removal of most frequent microplastic types and sizes in secondary effluent using Al_2_(SO_4_)_3_: choosing variables by a fuzzy Delphi method**

Nahid Azizi^1^, Meghdad Pirsaheb ^2,3^, Nematollah Jaafarzadeh Haghighifard^4^, Ramin Nabizadeh Nodehi^1*^

^1 Department of Environmental Health Engineering, School of Public Health, Tehran University of Medical Sciences, Tehran, Iran^

^2 Research Center for Environmental Determinants of Health (RCEDH), Health Institute, Kermanshah University of Medical Sciences, Kermanshah, Iran^

^3 Department of Environmental Health Engineering, Faculty of Health, Kermanshah University of Medical Sciences, Kermanshah, Iran^

^4 Department of Environmental Health Engineering, School of Public Health, Ahvaz Jundishapur University of Medical Sciences, Ahvaz, Iran^

^[Corresponding author. Tel: +98-912-289-2878 E-mail:^ [^rnabizadeh@gmail.com^](mailto:rnabizadeh@gmail.com)^]^

# Fuzzy Delphi

## validity and reliability

In this study, the validity and reliability of the questionnaire were investigated using common methods. First, the basis of the questionnaire design for the first phase of the research was a systematic review of the research literature and articles related to the identification of effective variables in the removal of microplastics by the coagulation process. Then, the questionnaire was given to the research team members for the initial test, so the defects and possible misinterpretations of the questions can be identified and corrected. After making the necessary corrections, the results showed that the experts had a mutual understanding of the subject and questions, which shows the validity of the questionnaire structure. Therefore, the questions were approved by the experts, which indicates the validity or reliability of the content of the questionnaire.

To check Cronbach's alpha value and the normality of the data, SPSS version 26 software was used. The Cronbach's alpha value of the first-course questionnaire is shown in Table S1-1. As can be seen, Cronbach's alpha value of the questionnaire (0.878) is higher than the value of 0.7, which confirms the reliability.

Table S 1- 1: Cronbach's alpha value

| Reliability Statistics | |
| --- | --- |
| Cronbach's Alpha | N of Items |
| 0.878 | 23 |

## Data normality

In this research, we investigated the normality of the data based on the amount of skewness and Kurtosis of the data (Table S1-2). As can be seen, the statistical values for the skewness and Kurtosis, (except for questions 11 and 29) are in the range of -2 and 2; therefore, there is normality in the data related to each question.

Table S 1- 2

| Descriptive Statistics | | | | | |
| --- | --- | --- | --- | --- | --- |
|  | N | Skewness | | Kurtosis | |
|  | Statistic | Statistic | Std. Error | Statistic | Std. Error |
| Q1 | 22 | .166 | .491 | -1.291 | .953 |
| Q2 | 22 | .000 | .491 | -.979 | .953 |
| Q3 | 22 | -.249 | .491 | -1.490 | .953 |
| Q4 | 22 | -1.003 | .491 | -.422 | .953 |
| Q5 | 22 | -.736 | .491 | -.459 | .953 |
| Q6 | 22 | -.776 | .491 | -.221 | .953 |
| Q7 | 22 | -.106 | .491 | -.806 | .953 |
| Q9 | 22 | -.654 | .491 | -.972 | .953 |
| Q10 | 22 | .289 | .491 | -.794 | .953 |
| Q11 | 22 | 1.825 | .491 | 3.442 | .953 |
| Q12 | 22 | -.142 | .491 | -1.104 | .953 |
| Q15 | 22 | -.694 | .491 | -.438 | .953 |
| Q16 | 22 | -.098 | .491 | .292 | .953 |
| Q17 | 22 | -.594 | .491 | .035 | .953 |
| Q18 | 22 | -.964 | .491 | -.122 | .953 |
| Q21 | 22 | .079 | .491 | -1.614 | .953 |
| Q22 | 22 | -.598 | .491 | -.123 | .953 |
| Q25 | 22 | -1.131 | .491 | .659 | .953 |
| Q26 | 22 | -.686 | .491 | -.614 | .953 |
| Q27 | 22 | -.193 | .491 | -.712 | .953 |
| Q28 | 22 | -1.144 | .491 | 1.806 | .953 |
| Q29 | 22 | -1.722 | .491 | 2.353 | .953 |
| Q32 | 22 | -.951 | .491 | .231 | .953 |
| Valid N (list wise) | 22 |  |  |  |  |

## Calculation of the fuzzy value of each of the research questions

After collecting the opinions of the experts, at this stage, based on the collected data, we calculated the fuzzy value of each of the questions (indices) as follows(Hsu, Lee et al. 2010, Liu 2013):

Assuming that the fuzzy value of each question is displayed as $\tilde{A_{j}}= (L_{j} . M_{j} . U_{j})$ and $L_{j}$ ، $M_{j}$, and $U_{j}$ are the lower, middle, and upper limit of this fuzzy number, they can be calculated as:

$$L_{j}=Min\left( x_{ij} \right) i=1. 2. \ldots. n j= 1. 2. \ldots. m$$

$$M_{j}={(\prod_{i=1}^{n.m} x_{ij})}^{\frac{1}{n}} i=1. 2. \ldots. n j= 1. 2. \ldots. m$$

$$U_{j}=Max\left( x_{ij} \right) i=1. 2. \ldots. n j= 1. 2. \ldots. m$$

$L_{j}$ : The lower limit of the fuzzy value of question j is equal to the smallest value assigned by the experts to question j.

$M_{j}$ : The mean value of the fuzzy value of question j is equal to the geometric mean of all expert opinions for question j.

$U_{j}$ : The higher limit of the fuzzy value of question j is equal to the largest value assigned by the experts to question j.

Then the fuzzy value obtained for each of the questions is de-fuzzed so that it is possible to compare and evaluate:

$$S_{j}=\frac{L_{j} + M_{j} + U_{j}}{3}$$

After calculating the determined De-fuzzy value of each of the questions, it is necessary to evaluate their importance.

If Sj≥r, it means that question j is of great importance.

If Sj<r, it means that question j is of little importance. Due to the low importance of these questions, they can be removed in the next courses of the study. Table S1-3 presents the fuzzy and De-fuzzy values of each of the questions of the first course.

Furthermore, eight open questions were asked at the end of the questions related to each index to get additional opinions of experts in the form of "If you have another opinion about the relevant index, enter your comments in the box below." And the two optional questions were asked to choose the type of microplastic and coagulant among the available items.

Table S 1- 3 Fuzzy and De-Fuzzy values related to each question

| Row | Questions | Fuzzy value | | | Sj | Significance |
| --- | --- | --- | --- | --- | --- | --- |
|  |  | Uj | Mj | Lj |  |  |
| 1 | What do you think about the placement of the coagulation process before the activated sludge? | 5 | 2.37 | 1 | 2.79 | Non-Significant |
| 2 | What do you think about the placement of the coagulation process after the activated sludge? | 5 | 3.35 | 2 | 3.45 | Significant |
| 3 | What do you think about the placement of the coagulation process before and after the activated sludge and compare the results? | 5 | 2.82 | 1 | 2.94 | Non-Significant |
| 4 | What do you think about adding different concentrations of microplastics to wastewater as an independent variable? | 5 | 3.22 | 1 | 3.07 | Significant |
| 5 | What do you think about using the results of microplastic concentration measured in wastewater treatment plants? | 5 | 3.42 | 1 | 3.14 | Significant |
| 6 | What do you think about the use of data in articles on microplastic removal by coagulation as microplastic concentrations? | 5 | 3.35 | 1 | 3.12 | Significant |
| 7 | What do you think about using different types of microplastics (all sex types identified in wastewater treatment plants) as variables? | 5 | 2.87 | 1 | 2.95 | Non-Significant |
| 8 | What do you think about considering only three types of microplastics (PE, PS, and PA) that conventional wastewater treatment is unable to remove significantly? | 5 | 3.20 | 1 | 3.06 | Significant |
| 9 | What do you think about considering only the two types of PE and PS used in the previous articles about microplastic removal by coagulation? | 5 | 2.61 | 1 | 2.87 | Non-Significant |
| 10 | What do you think about using only one type of microplastic as an indicator of total microplastics? | 5 | 1.72 | 1 | 2.57 | Non-Significant |
| 11 | What do you think about using all size classifications (all 7 categories A to G) for microplastics as a variable? | 5 | 2.96 | 1 | 2.99 | Non-Significant |
| 12 | What do you think about using microplastic sizes that are not significantly removed at different steps of conventional wastewater treatment plants (sizes 10 to 600 micrometers related to categories A to D) as variables? | 5 | 3.75 | 2 | 3.58 | Significant |
| 13 | What do you think about using two categories of less than and greater than 500 micrometers for microplastic removal by previous coagulation articles? | 5 | 2.83 | 1 | 2.94 | Non-Significant |
| 14 | What do you think about the new size classification based on previous studies for experiments? | 5 | 3.11 | 1 | 3.03 | Significant |
| 15 | What do you think about using wastewater as a matrix and adding microplastics with specified characteristics? | 5 | 3.48 | 1 | 3.16 | Significant |
| 16 | What do you think about the use of tap water as a matrix and adding microplastics with specified characteristics? | 5 | 2.65 | 1 | 2.88 | Non-Significant |
| 17 | What do you think about using AlCl3:6H2O coagulant? | 5 | 3.33 | 1 | 3.11 | Significant |
| 18 | What do you think about using Al(OH)3 coagulant? | 5 | 3.35 | 1 | 3.12 | Significant |
| 19 | What do you think about using FeCl3 coagulant? | 5 | 3.29 | 1 | 3.09 | Significant |
| 20 | What do you think about using Al2(SO4)3 coagulant? | 5 | 2.84 | 1 | 2.94 | Non-Significant |
| 21 | What do you think about using PAC coagulant? | 5 | 3.63 | 1 | 3.21 | Significant |
| 22 | What do you think about using one or more of the coagulants mentioned in the previous questions and comparing the results? | 5 | 3.98 | 1 | 3.32 | Significant |
| 23 | What do you think about doing enhanced coagulation instead of traditional coagulation? | 5 | 3.53 | 1 | 3.17 | Significant |

No new index has been introduced by the experts in the first course, so no new question will be added to the second questionnaire. On the other hand, to design the second questionnaire, the questions that are known as non-significant in the first questionnaire should be removed.

## Preparing second course of questionnaire

In this step, according to the results obtained from the first course, we design the second questionnaire. According to the amount of fuzzy value, we can ignore the questions that are not important enough in the research. Also, if the experts have suggested a new variable, we will add to the previous questions and continue this pattern until all the questions identify as important.

According to Table 2, all questions of the second questionnaire are effective in the process of microplastic removal through coagulation. On the other hand, as in the previous round, no new index has been proposed by the experts. Therefore, based on these results, we have reached the goal of the research, which is to identify the effective variables for removing microplastics through coagulation.

**References**

Hsu, Y.-L., C.-H. Lee and V. B. Kreng (2010). "The application of Fuzzy Delphi Method and Fuzzy AHP in lubricant regenerative technology selection." Expert Systems with Applications **37**(1): 419-425.

Liu, W.-K. (2013). "Application of the fuzzy delphi method and the fuzzy analytic hierarchy process for the managerial competence of multinational corporation executives." International Journal of e-Education, e-Business, e-Management and e-Learning **3**(4): 313.
