# Supplementary material for: Removal of most frequent microplastic types and sizes in secondary effluent using Al2(SO4)3: choosing variables by a fuzzy Delphi method
Source: Sci Rep. 2023 Nov 25;13:20718. doi: 10.1038/s41598-023-47803-4 (PMC10676421; doi:10.1038/s41598-023-47803-4)
Supplement: Supplementary file 2 — Supplementary Information 2. [file 41598_2023_47803_MOESM2_ESM.docx]

**Supplementary file 2**

**Removal of most frequent microplastic types and sizes in secondary effluent using Al_2_(SO_4_)_3_: choosing variables by a fuzzy Delphi method**

Nahid Azizi^1^, Meghdad Pirsaheb ^2,3^, Nematollah Jaafarzadeh Haghighifard^4^, Ramin Nabizadeh Nodehi^1*^

^1 Department of Environmental Health Engineering, School of Public Health, Tehran University of Medical Sciences, Tehran, Iran^

^2 Research Center for Environmental Determinants of Health (RCEDH), Health Institute, Kermanshah University of Medical Sciences, Kermanshah, Iran^

^3 Department of Environmental Health Engineering, Faculty of Health, Kermanshah University of Medical Sciences, Kermanshah, Iran^

^4 Department of Environmental Health Engineering, School of Public Health, Ahvaz Jundishapur University of Medical Sciences, Ahvaz, Iran^

^[Corresponding author. Tel: +98-912-289-2878 E-mail:^ [^rnabizadeh@gmail.com^](mailto:rnabizadeh@gmail.com)^]^

Table S 2- 1 List of variables based on review of previous studies

| Row | Indices | Options | value |
| --- | --- | --- | --- |
| 1 | location of coagulation process | Before activated sludge/after activated sludge | - |
| 2 | MP concentration | Base of WWTP effluent/base of laboratory experiments | 7863 Number per liter |
|  |  |  | <500,>500 mg/L |
| 3 | MP type | MP types in WWTP/ MP types used in laboratory experiments | - |
| 4 | MP size | MP sizes in WWTP/ MP sizes used in laboratory experiments | <600 μm  <500,>500 μm |
| 5 | water matrix | Tap water/ wastewater | - |
| 6 | coagulant type | AlCl3:6H2O/ Al(OH)3/ FeCl3/ Al2(SO4)3/PAC | - |
| 7 | enhanced coagulation | Use co-coagulant/ non-use co- coagulant | - |

Table S 2- 2 Experimental design

| **Polyethylene** | | | **Polystyrene** | | | **Polyamide** | | |
| --- | --- | --- | --- | --- | --- | --- | --- | --- |
| **Run** | **MP Size** | **Al2SO4 (mg/l)** | **Run** | **MP Size** | **Al2SO4 (mg/l)** | **Run** | **MP Size** | **Al2SO4 (mg/l)** |
| 1 | Small | 5 | 23 | Small | 5 | 45 | Small | 5 |
| 2 | Small | 10 | 24 | Small | 10 | 46 | Small | 10 |
| 3 | Small | 20 | 25 | Small | 20 | 47 | Small | 20 |
| 4 | Small | 30 | 26 | Small | 30 | 48 | Small | 30 |
| 5 | Small | 40 | 27 | Small | 40 | 49 | Small | 40 |
| 6 | Small | 50 | 28 | Small | 50 | 50 | Small | 50 |
| 7 | Small | 60 | 29 | Small | 60 | 51 | Small | 60 |
| 8 | Small | 70 | 30 | Small | 70 | 52 | Small | 70 |
| 9 | Small | 80 | 31 | Small | 80 | 53 | Small | 80 |
| 10 | Small | 90 | 32 | Small | 90 | 54 | Small | 90 |
| 11 | Small | 100 | 33 | Small | 100 | 55 | Small | 100 |
| Blank1 | Small | 0 | Blank3 | Small | 0 | Blank5 | Small | 0 |
| 12 | Large | 5 | 34 | Large | 5 | 56 | Large | 5 |
| 13 | Large | 10 | 35 | Large | 10 | 57 | Large | 10 |
| 14 | Large | 20 | 36 | Large | 20 | 58 | Large | 20 |
| 15 | Large | 30 | 37 | Large | 30 | 59 | Large | 30 |
| 16 | Large | 40 | 38 | Large | 40 | 60 | Large | 40 |
| 17 | Large | 50 | 39 | Large | 50 | 61 | Large | 50 |
| 18 | Large | 60 | 40 | Large | 60 | 62 | Large | 60 |
| 19 | Large | 70 | 41 | Large | 70 | 63 | Large | 70 |
| 20 | Large | 80 | 42 | Large | 80 | 64 | Large | 80 |
| 21 | Large | 90 | 43 | Large | 90 | 65 | Large | 90 |
| 22 | Large | 100 | 44 | Large | 100 | 66 | Large | 100 |
| Blank2 | Large | 0 | Blank4 | Large | 0 | Blank6 | Large | 0 |

Table S 2- 3 Characteristics of expert panel members

| **Educational and research activities (years)** | | | | **Number** | **Science ranking** |
| --- | --- | --- | --- | --- | --- |
| **>20** | **10-20** | **5-10** | **<5** |  |  |
| 3 | 2 | - | - | 5 | Professor |
| - | 5 | 1 | - | 6 | Associate Professor |
| 2 | - | 5 | 4 | 11 | Assistant Professor |

Table S 2- 4 Fuzzy and De-Fuzzy values related to each question

| **Row** | **Questions** | **Fuzzy value** | | | **Significance** |
| --- | --- | --- | --- | --- | --- |
|  |  | **Uj** | **Mj** | **Lj** |  |
| 1 | What do you think about placing the coagulation process as a tertiary treatment (after activated sludge)? | 5 | 3.29 | 1.00 | Significant |
| 2 | What do you think about the use of results related to measured amounts of microplastics in previous articles? | 5 | 3.77 | 2.00 | Significant |
| 3 | What is your opinion about the use of three types of microplastics (PE, PS, and PA) that conventional wastewater treatment is unable to significantly remove? | 5 | 3.94 | 2.00 | Significant |
| 4 | What is your opinion on the use of microplastic sizes (10-600 μm) that are not significantly removed at different steps of conventional wastewater treatment plants and the classification of this range based on available meshes? | 5 | 3.58 | 2.00 | Significant |
| 5 | What do you think about using wastewater from secondary treatment and adding microplastics with specified characteristics? | 5 | 3.59 | 2.00 | Significant |
| 6 | What is your opinion about using all the available coagulants and doing a pre-test to determine the appropriate coagulant to use in the tests? | 5 | 3.38 | 1.00 | Significant |
| 7 | What do you think about performing enhanced coagulation instead of traditional coagulation for experiments? | 5 | 3.80 | 1.00 | Significant |

Table S 2- 5 ANOVA results for difference in removal efficiency of microplastic type and size

| Variable |  | Df | Sum Sq | Mean sq | F value | Pr(>F) |
| --- | --- | --- | --- | --- | --- | --- |
| MPs size | Size | 1 | 31799 | 31799 | 59.32 | 1.08×10^-10 ***^ |
|  | Residuals | 64 | 34307 | 536 | - | - |
| MPs type | Type | 2 | 17579 | 8790 | 11.41 | 5.9×10^-5***^ |
|  | Residuals | 63 | 48527 | 770 | - | - |

^Significant codes: 0 ‘***’ 0.001 ‘**’ 0.01 ‘*’ 0.05 ‘.’ 0.1 ‘’ 1,^

#####
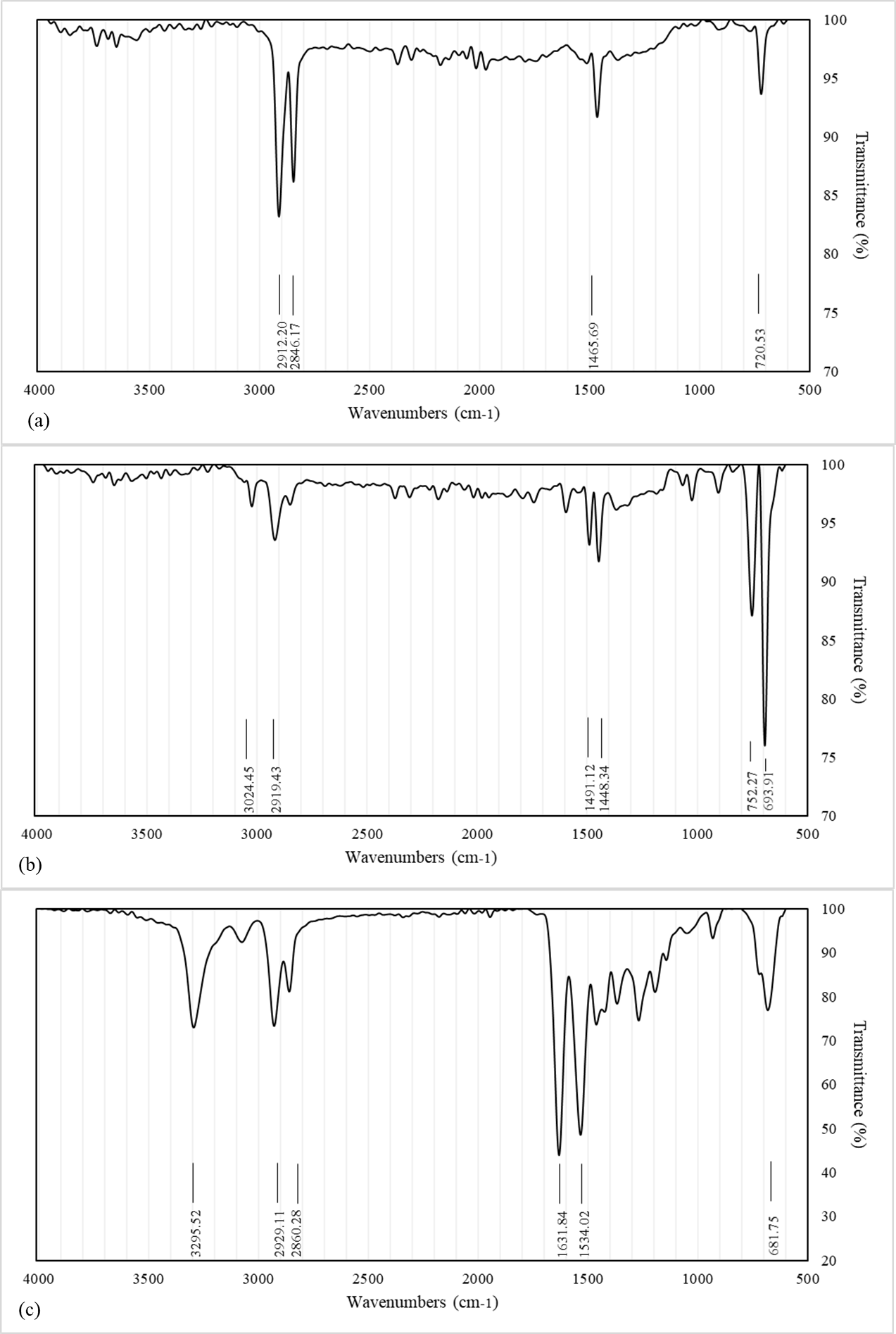


Figure S 2- 1: FTIR a)PE, c) PS, e)PA compare to graph by (Jung, Horgen et al. 2018)

Figure S 2- 2 Average microplastic removal in different coagulant dose in case of microplastic types


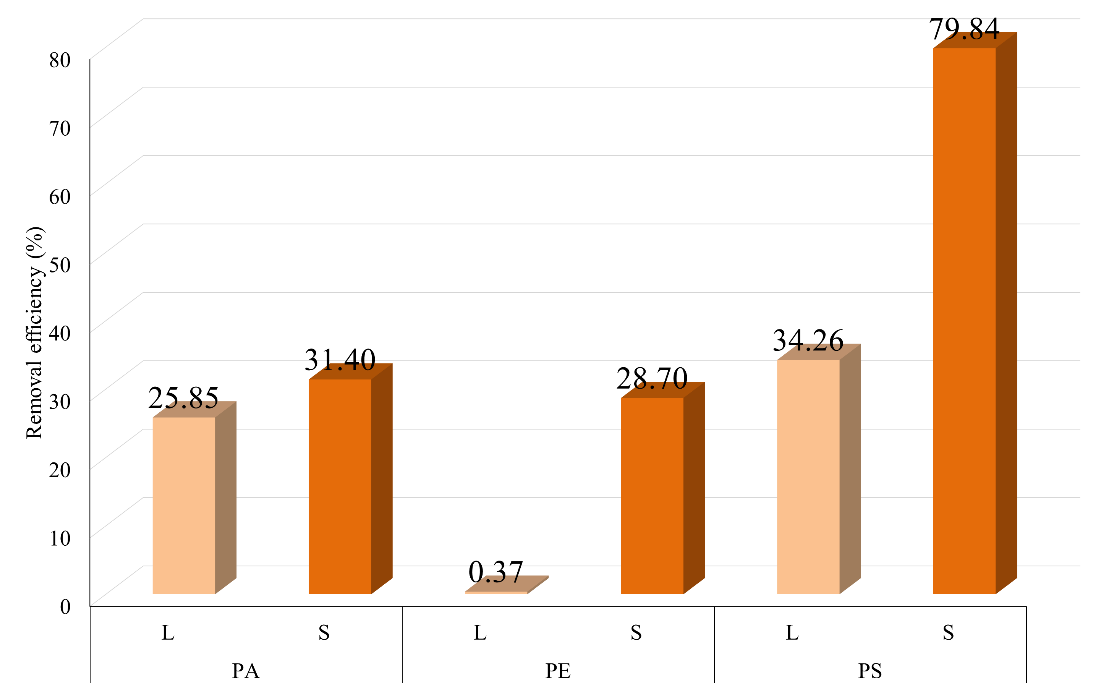


Figure S 2- 3 Removal efficiency in Blanks in case of microplastics type and size

Table S 2- 6 Balanced one-way analysis of variance power calculation for microplastic type and size

|  | **Microplastic type** | **Microplastic size** |
| --- | --- | --- |
| **groups** | 3 | 2 |
| **n** | 22 | 33 |
| **between. Variance** | 250.49 | 706.43 |
| **within. Variance** | 1001.96 | 2825.74 |
| **sig. level** | 0.05 | 0.05 |
| **power** | 83.4% | 80.7% |

^NOTE: n is number in each group^

Table S 2- 7 post-hoc analysis (TukeyHSD) for MPs removal efficiency for different MP types

| MP types | diff | lwr | upr | p adj |
| --- | --- | --- | --- | --- |
| PE-PA | -39.6109 | -59.697 | -19.5248 | 3.81E-05^*^ |
| PS-PA | -24.4746 | -44.5606 | -4.38846 | 0.013104^*^ |
| PS-PE | 15.13636 | -4.94972 | 35.22245 | 0.174983 |

**References**

Jung, M. R., F. D. Horgen, S. V. Orski, V. Rodriguez, K. L. Beers, G. H. Balazs, T. T. Jones, T. M. Work, K. C. Brignac and S.-J. Royer (2018). "Validation of ATR FT-IR to identify polymers of plastic marine debris, including those ingested by marine organisms." Marine Pollution Bulletin **127**: 704-716.
