# Supplementary material for: Removal of most frequent microplastic types and sizes in secondary effluent using Al2(SO4)3: choosing variables by a fuzzy Delphi method
Source: Sci Rep. 2023 Nov 25;13:20718. doi: 10.1038/s41598-023-47803-4 (PMC10676421; doi:10.1038/s41598-023-47803-4)
Supplement: Supplementary file 3 — Supplementary Information 3. [file 41598_2023_47803_MOESM3_ESM.docx]

**Supplementary file 3**

**Removal of most frequent microplastic types and sizes in secondary effluent using Al_2_(SO_4_)_3_: choosing variables by a fuzzy Delphi method**

Nahid Azizi^1^, Meghdad Pirsaheb ^2,3^, Nematollah Jaafarzadeh Haghighifard^4^, Ramin Nabizadeh Nodehi^1*^

^1 Department of Environmental Health Engineering, School of Public Health, Tehran University of Medical Sciences, Tehran, Iran^

^2 Research Center for Environmental Determinants of Health (RCEDH), Health Institute, Kermanshah University of Medical Sciences, Kermanshah, Iran^

^3 Department of Environmental Health Engineering, Faculty of Health, Kermanshah University of Medical Sciences, Kermanshah, Iran^

^4 Department of Environmental Health Engineering, School of Public Health, Ahvaz Jundishapur University of Medical Sciences, Ahvaz, Iran^

^[Corresponding author. Tel: +98-912-289-2878 E-mail:^ [^rnabizadeh@gmail.com^](mailto:rnabizadeh@gmail.com)^]^

# Conversion of microplastic abundance to weight value

The concentration of microplastics in wastewater in all the articles that were systematically studied was based on the number of microplastics per liter. In addition, according to the Delphi research results, it was concluded that reported microplastics concentrations in the effluent of wastewater treatment plants by previous articles should be used in the experiment. Therefore, to facilitate the experiment and avoid wasting time counting microplastics, the weight value of different microplastic types and sizes was modeled. Then, the weight corresponding to the number of microplastics was calculated using the obtained model. There are six models for three types of microplastics, each with two size ranges (PE, PS, and PA, each in two size range less than 125 and 300 to 600 micrometers) (Table S3-1, S3-2, S3-3).

To create the mentioned model for each size of a specific type of microplastic, the first five different weights of microplastic were weighted. Due to the low concentration of microplastics and the high accuracy of the scale (minimum weight range of 1x10-5 g (CP225D, SARTORIUS AG GÖTTINGEN, Germany)), each weight was measured five times, and the average was used. Then, the desired weight was counted three times under the loupe device (LEICA MS 5), and the average number obtained was used in the model. Finally, the graph and equation of the desired model were obtained by Excel software.

Table S 3- 1: Polyethylene microplastics converting count to weight model for <125 , 300-600 micrometer sizes

|  | PE 300-600 Micrometer | | | PE<125 Micrometer | | |
| --- | --- | --- | --- | --- | --- | --- |
|  | Weight(mg) | Count | Weight model(mg) | weight | count | weight model(mg) |
| 1 | 0 | 0 | -0.35 | 0 | 0 | 0.0086 |
| 2 | 1.412 | 47.33333 | 0.407333333 | 1.34 | 2336 | 1.1766 |
| 3 | 4.7775 | 183.6667 | 2.588666667 | 2.896333 | 6420 | 3.2186 |
| 4 | 9.85 | 683.6667 | 10.58866667 | 6.132 | 11639 | 5.8281 |
| 5 | 19.736 | 1298.333 | 20.42333333 | 9.028333 | 18059 | 9.0381 |
| 6 | 50.408 | 3475 | 55.25 |  |  |  |
| 7 | 102.016 | 6196 | 98.786 |  |  |  |
| max | in 1 liter | 7863 | **125.458** | in 1 liter | 7863 | **3.9401** |

Table S 3- 2: Polystyrene microplastics converting count to weight model for <125 , 300-600 micrometer sizes

|  | PS 300-600 Micrometer | | | PS<125 Micrometer | | |
| --- | --- | --- | --- | --- | --- | --- |
|  | Weight(mg) | Count | Weight model(mg) | Weight(mg) | count | weight model(mg) |
| 1 | 0 | 0 | 1.4681 | 0 | 0 | 0.4267 |
| 2 | 4.648 | 207 | 4.4489 | 2.28 | 2470 | 1.6617 |
| 3 | 23.22558 | 1406 | 21.7145 | 6.838 | 15219 | 8.0362 |
| 4 | 53.1325 | 3563 | 52.7753 | 10.254 | 22827 | 11.8402 |
| 5 | 100.336 | 6880 | 100.5401 | 12.534 | 25297 | 13.0752 |
| max | in 1 liter | 7863 | **114.6953** | in 1 liter | 7863 | **4.3582** |

Table S 3- 3: Polyamide microplastics converting count to weight model for <125 , 300-600 micrometer sizes

|  | PA 300-600 Micrometer | | | PA<125 Micrometer | | |
| --- | --- | --- | --- | --- | --- | --- |
|  | Weight(mg) | Count | Weight model (mg) | Weight(mg) | count | weight model(mg) |
| 1 | 0 | 0 | -1.1968 | 0 | 0 | -0.0805 |
| 2 | 1.86 | 186 | 0.161 | 0.255 | 2983 | 0.5161 |
| 3 | 4.548 | 602 | 3.1978 | 0.535 | 4648 | 0.8491 |
| 4 | 10.7225 | 2082 | 14.0018 | 1.135 | 6964 | 1.3123 |
| 5 | 23.055 | 3523 | 24.5211 | 1.39 | 9947 | 1.9089 |
| 6 | 34.9375 | 5380 | 38.0772 |  |  |  |
| 7 | 60.255 | 7995 | 57.1667 |  |  |  |
| max | in 1 liter | 7863 | **56.2031** | in 1 liter | 7863 | **1.4921** |
